# Visuomotor Adaptation Deficits in Patients with Essential Tremor

**DOI:** 10.1007/s12311-022-01474-5

**Published:** 2022-09-09

**Authors:** Laura Bindel, Christoph Mühlberg, Victoria Pfeiffer, Matthias Nitschke, Annekatrin Müller, Mirko Wegscheider, Jost-Julian Rumpf, Kirsten E. Zeuner, Jos S. Becktepe, Julius Welzel, Miriam Güthe, Joseph Classen, Elinor Tzvi

**Affiliations:** 1 Department of Neurology, Leipzig University, Liebigstraße 20, 04103 Leipzig, Germany; 2 Department of Neurology, University of Lübeck, 23562 Lübeck, Germany; 3 Department of Neurology, Kiel University, 24105 Kiel, Germany; 4 Syte Institute, 20354 Hamburg, Germany

**Keywords:** Essential tremor, Movement disorders, Motor learning, Visuomotor adaptation, Cerebellum

## Abstract

Essential tremor (ET) is a progressive movement disorder whose pathophysiology is not fully understood. Current evidence supports the view that the cerebellum is critically involved in the genesis of the tremor in ET. However, it is still unknown whether cerebellar dysfunction affects not only the control of current movements but also the prediction of future movements through dynamic adaptation toward a changed environment. Here, we tested the capacity of 28 patients with ET to adapt in a visuomotor adaptation task known to depend on intact cerebellar function. We found specific impairments in that task compared to age-matched healthy controls. Adaptation to the visual perturbation was disrupted in ET patients, while de-adaptation, the phase after abrupt removal of the perturbation, developed similarly to control subjects. Baseline tremor-independent motor performance was as well similar to healthy controls, indicating that adaptation deficits in ET patients were not rooted in an inability to perform goal-directed movements. There was no association between clinical severity scores of ET and early visuomotor adaptation abilities. These results provide further evidence that the cerebellum is dysfunctional in ET.

**Supplementary Information:**

The online version contains supplementary material available at 10.1007/s12311-022-01474-5.

## Introduction

Essential tremor (ET) is a common and potentially disabling progressive movement disorder that may induce significant burden for affected patients [1]. The cerebellum plays an important role in the pathophysiological processes underlying ET, as evident by neuropathological and neuroimaging studies [2–10]. Clinical evidence pointing to cerebellar dysfunction in ET includes the late-stage emergence of intention tremor, of gait and balance abnormalities, oculomotor dysfunctions and eye-hand coordination deficits which are cardinal signs of cerebellar pathology [11]. Despite the absence of marked cerebellar degeneration in pathological studies [12, 13], in vivo imaging studies have demonstrated loss of white and grey matter in cerebellar volume, albeit in variable cerebellar sub-regions [3, 14-19]. Pathophysiological studies of tremor generation in ET patients suggest that excessive oscillations in the cortico-thalamo-cerebellar network could underlie tremor manifestation [20–22]. Increased tremor-related activity in cerebellar lobules IV/V/VIII [15, 23] and normalization of cerebellar hyperactivity through thalamic stimulation (DBS) [24] further support this hypothesis. In addition, decreased functional connectivity between dentate nucleus and cortical areas, thalamus, and cerebellar cortex [25] indicates cerebellar involvement in ET. Overall, these studies suggest that cerebellar dysfunction, which also affects interactions with cortical and other subcortical regions, is involved in the pathophysiology of ET.

In the healthy cerebellum, sensorimotor information from cerebral cortex, as well as from spinal cord, is integrated to enable coordinated movements and to adjust and update error signals [26]. The latter functionality has positioned the cerebellum as a key structure in the process of motor skill learning and, more specifically, visuomotor adaptation, which requires motor commands to be adjusted through trial-and-error, to fit a pattern of visual stimuli [27]. Structural injury of the cerebellum, as present in patients with cerebellar degeneration or cerebellar stroke, impairs adaptation to a visual perturbation [28–32], including prism adaptation [33, 34], and to an external force field imposed on the movements [35, 36]. Furthermore, eyeblink conditioning, a paradigm in which an eyeblink is associatively bound to a conditioned stimulus as a result of repeated pairings with an unconditioned stimulus, was impaired in patients with cerebellar stroke [37, 38], and cerebellar degeneration [39, 40].

Eyeblink conditioning and prism adaptation are also compromised in ET [41, 42], which agrees with the assumption of cerebellar dysfunction in ET. However, it is not known whether ET can also affect motor plasticity in effector systems expressing tremor. To this end, we investigated whether visuomotor reach adaptation is impaired in a cohort of mildly to moderately affected ET patients. This paradigm tests upper-limb center-out movements in the presence or absence of a visual perturbation on a computer screen. By showing impairment of visuomotor adaptation in ET patients, the present study provides further evidence of cerebellar involvement in ET.

## Materials and Methods

### Cohort

All participants provided written informed consent before experiments commenced. Participants’ consent was obtained according to the Declaration of Helsinki and the study was approved by the Ethics Committees of Leipzig University (279/20-ek), Kiel University (B 264/21), and University of Lübeck.

Assuming an effect size of 0.6, and 0.8 power, we calculated a sample size of 36 subjects per group which is required to find evidence for a difference in means between two independent groups using a *t*-test. We therefore recruited 38 ET patients through self-support groups, departments of neurology in Leipzig and Kiel, as well as outpatient clinics of Leipzig University hospital and Lübeck University hospital. Six patients who received primidone (see supplementary Table 1) were excluded post hoc due to a possible effect of primidone (barbiturates) on cerebellar function. In the supplementary materials, Sects. 1–5, we present the analysis of the entire cohort, including the six excluded patients receiving primidone, as well as the six healthy controls matched to these patients. Four additional patients were excluded due to false diagnosis of ET and/or technical failure during the experiment, resulting in a final cohort of 28 ET patients. To this patient cohort, we age-matched 28 healthy controls who were patients’ spouses or partners or recruited through databases of Leipzig University.

Participants were non-smokers, had no history of alcohol or drug abuse, had no professional musical or typing experience, and had normal or corrected to normal vision. No upper age limit was set for this study. General cognitive abilities were tested using the Montreal Cognitive Assessment (MoCA) [43]. Those who scored ≥ 23 points were eligible to participate (higher scores reflect better cognitive abilities). Note that while 23–26 points might indicate mild cognitive impairment (MCI), a cutoff at score of 26 leads to a higher rate of false-positive of MCI especially for those of older age and/or lower education [44–47]. Both right- and left-handed participants were included. We determined handedness using the Edinburgh Handedness Inventory (EHI) [48]. The visuomotor adaptation task was performed with the dominant hand as confirmed by the EHI-Score. In our cohort, 26 patients and 26 controls were right-handed. Two patients and two controls were ambidextrous, but only one patient completed the task with the left hand. We used the Beck Depression Inventory (BDI) to screen for depression [49]. Included participants had ≤ 19 points (lower scores corresponding to no depression or only mild depressive symptoms). Participants with other internal, orthopedic, neurological, or psychiatric disease that could influence performance in the task were excluded.

The diagnosis “essential tremor” was made by expert neurologists (JJR, JC, AM, MN, JB) prior to study participation. Included ET patients had mild to moderate upper-limb tremor as evaluated by “The Essential Tremor Rating Assessment Scale” (TETRAS) including the “Activities of Daily Living” Subscale and the “Performance” Subscale [50]. We also evaluated possible cerebellar symptoms using the Scale for the Assessment and Rating of Ataxia (SARA) [51]. Two investigators rated the entire ET cohort.

### Clinical Characteristics of the Essential Tremor Cohort

Age, sex, disease duration, current medication, alcohol responsivity, and cognitive functions assessed with MoCA were evaluated as clinical characteristics (Supplementary Table 1). Mean age was not statistically different between the 28 ET patients and the matched healthy controls (ET: 57.9 ± 21.0 years; CON: 57.5 ± 21.1 years; *P* = 0.5). In addition, cognitive abilities assessed with MoCA were not statistically different between groups (ET: 27.7 ± 2.0 points; CON: 28.1 ± 1.7; *P* = 0.36). Essential tremor patients had a mean disease duration of 25.2 ± 20.3 years (*n* = 26, information on disease duration of two patients was missing). Eight patients were on symptomatic treatment with propranolol or topiramate at the time of testing. Twenty patients took no medication to treat ET. The mean tremor severity as measured by the total TETRAS score was 30.6 ± 12.8 indicating a mild to moderate tremor severity.

### Visuomotor Adaptation Task: Experimental Design

**Fig. 1:**
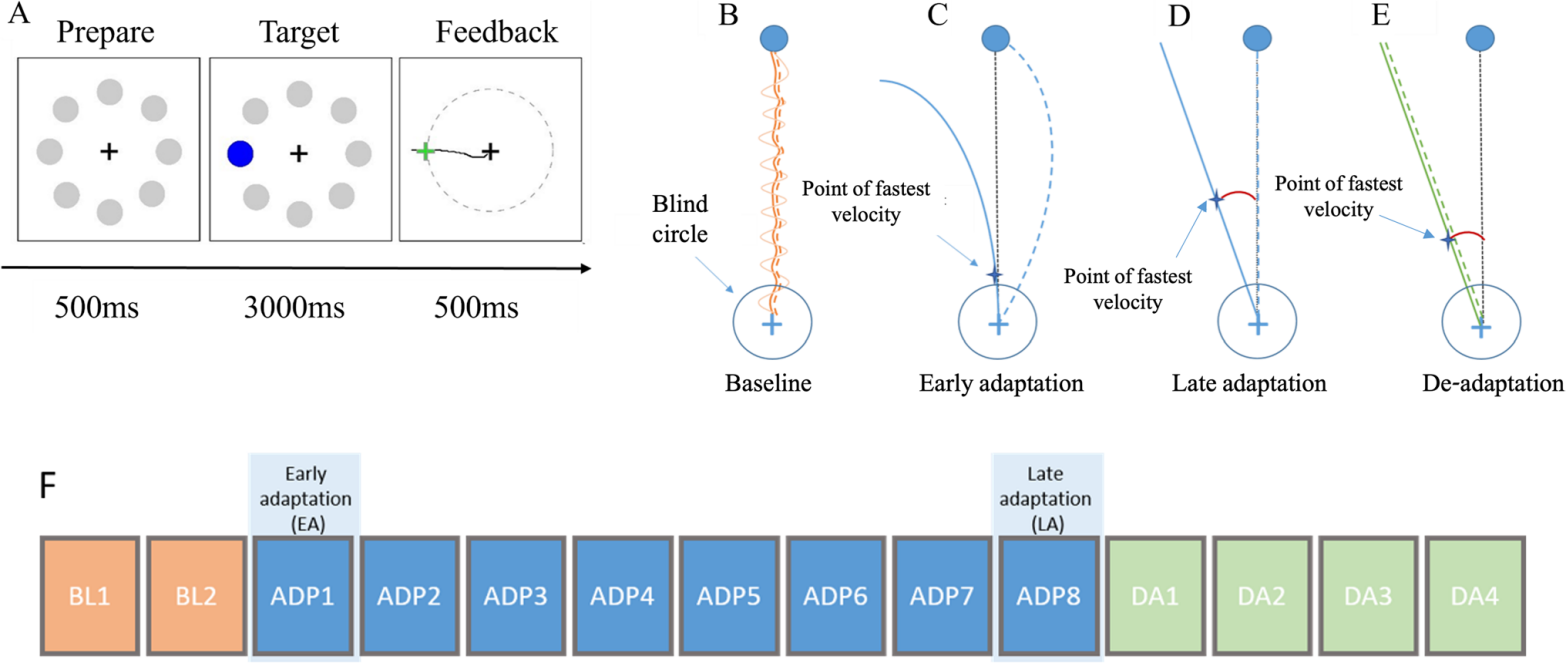
The visuomotor adaptation task. **A** Trial timeline. Each trial began with 500 ms presentation of 8 possible targets—grey circles evenly distributed around a central black cross (“Prepare”). Next, the current target was marked in blue, signaling participants to start the movement with the digital pen (“Target”). This movement was projected to the screen. When subjects crossed the invisible circle connecting the edges of all targets, feedback was given using a green cross at the crossing point (“Feedback”). **B**–**E** Illustration of participants’ movements during the different task conditions. Dashed lines present the movement displayed on the screen. Point of fastest velocity could be traced to any location along the movement line between the “blind circle” and the target. **B** Baseline phase. Light orange: original movement; dark orange: smoothed movement. The smoothing was performed in all conditions although it is here presented for simplicity during baseline only. **C** Early adaptation. Blue line: original movement; dashed line: 30° rotated movement visualized on the screen; minimal to no adaptation is indicated by the small angular error between point of fastest velocity and the connection line of cross and target. Here we present an example of a corrective movement which we instructed the patients to *avoid*. **D** Late adaptation. Blue line: original movement; the movement on the screen is similar to the line connecting the cross and the target. Enhanced adaptation is indicated by the large angular error between point of fastest velocity and the connecting line. **E** De-adaptation. Large angular error between point of fastest velocity and the connecting line. **F** The time course of the task. Each block contained 24 trials

Participants performed a visuomotor adaptation task [52], while sitting comfortably in front of a computer screen. At the beginning of each trial, eight grey circles appeared on the screen in one of eight possible positions arrayed around a central cross, equally distributed at a distance of 300 pixels, every 45°. Next, one of the eight circles was marked as a blue target, and participants had to move from the central cross toward the target moving a digital pen on a digital tablet (Wacom Intuos Pro L, Wacom, Kazo, Japan). The targets were presented pseudo-randomly such that every set of eight consecutive trials included one of each target positions. The movement on the tablet was represented as a cursor on the screen. All participants were instructed to perform straight, “shooting through” hand movements. Visual feedback from the moving hand was prevented. The pen position was sampled at 60 Hz (adjacent sampling points recorded every ~ 17 ms). Movement onset was defined as the first time point a deviation of more than five-pixel was detected between adjacent sampling points in either direction. The end of the movement was defined as the time point, when the cursor crossed an invisible circle connecting the edges of all targets. At that moment, all other stimuli disappeared, and feedback was given as a green cross at movement endpoint for 500 ms (Fig. 1A). The cursor was always visible on the screen. Participants were instructed to perform movements rapidly. To encourage participants to perform faster movements, a bar at the bottom of the screen visualized the progression of time during the trial. After 1.5 s, the bar turned red to indicate slow performance of the movement. If the subjects did not reach the target within 3 s, the trial was discarded, and the experiment proceeded with the next trial. The next trial started 500 ms after participants moved the cursor back to the center cross. The task was designed using Psychtoolbox-3 (Brainard, 1997) operating on MATLAB R2019b (Mathworks®).

Over the course of the experiment, a 30° clockwise visuomotor perturbation of the cursor movement on the screen was introduced abruptly. Subjects were not informed about this perturbation prior to the experiment. The experiment was therefore divided into three phases: the baseline (BL) phase in which subjects executed simple center-target movements without perturbation (Fig. 1B), the adaptation (ADP) phase in which participants adapted their movement to the 30° clockwise perturbation of the cursor movement on the screen (Fig. 1C–D), and the de-adaptation (DA) phase that followed abrupt removal of the perturbation (Fig. 1E). In total, the experiment consisted of 14 blocks, with 24 trials each, and ten seconds breaks between the blocks. The BL condition consisted of two blocks (BL1, BL2), the ADP consisted of eight blocks (ADP1-ADP8) and the DA consisted of four blocks (DA1-DA4) (Fig. 1F). The task was designed to last approximately 30 min. Prior to the main experiment, participants performed a short trial run to familiarize themselves with the task. The trial run contained eight trials without perturbation as well as eight trials with random visual perturbations between -30° and 30° clockwise.

### Data Analysis

Analyses of visuomotor adaptation were performed using custom-made scripts in MATLAB. The first trial of each block was excluded from further analyses to rule out a potential interference by the break. Adaptation in each trial was operationally defined as the angular error between a straight line connecting the center cross and the target, and a line connecting the center cross and the position of the cursor at peak velocity (Fig. 1C1 & C2). To accurately calculate the angular error in the ET cohort, we used a 10-sample moving average filter to remove the tremor-component from the movement (see illustration in Fig. 1B). A negative angular error describes movements performed counterclockwise during ADP to counteract the 30° clockwise visual rotation in order to successfully hit the target. A positive angular error describes movements performed at clockwise rotation. We defined trials as outliers if the angular error exceeded 60° in any direction. To assess visuomotor adaptation, we defined three adaptation indices (AI) in which the angular error was normalized to the baseline (“early adaptation,” AI1 and “late adaptation,” AI2), or to the final stage of adaptation (“early de-adaptation,” AI3): AI1 was defined as the difference between the median angular error at the first adaptation block and the median angular error at the second baseline block (ADP1–BL2). AI2 was defined as the median angular error difference between the last adaptation block and the second baseline block (ADP8–BL2). AI3 was defined as the difference between the median angular error of the first de-adaptation block and the last adaptation block (DA1–ADP8). This strategy allowed us to evaluate individual adaptation compared to the baseline performance. We decided against comparing the last adaptation block to the first adaptation block (ADP8–ADP1) because this would falsely attribute poor adaptation to subjects who adapt very well already early on. Note that values for AI3 were positive based on the strategy of calculation. This means, larger (absolute) values correspond to better performance during the experiment.

Reaction times (RT) and movement times (MT) were analyzed to assess motor performance. Reaction time was defined as the interval between the appearance of the visual stimulus (Fig. 1A, “Target”) and movement onset (defined above), and MT was defined as the period between movement onset and the time in which the participant reached the outer boundary of the invisible circle connecting the targets (Fig. 1A, “Feedback”). Trials in which MT exceeded 2.7 standard deviations (SD) from the individual mean MT (~ 1% of all data) were excluded. RT-based trial exclusion was performed similarly.

Finally, to assess movement dynamics, we recorded the time interval between RT and the time at peak velocity in each trial.

### Statistical Analyses

Statistical analyses were performed on the median angular error using a mixed ANOVA with factors Group (ET, CON) and Block to analyze adaptation dynamics within the adaptation and the de-adaptation phase. The Wilcoxon-ranked sum test was used to compare the median angular error between groups and across the three AIs. RT and MT were analyzed similarly. In addition, we used Spearman correlation to analyze dependencies between clinical characteristics of ET and behavioral parameters of the visuomotor adaptation task.

### Data Availability

Personalized data are protected by data privacy statements signed by all subjects. Anonymized behavioral data can be made available upon reasonable request.

## Results

### Visuomotor Adaptation Is Impaired in Essential Tremor Patients

**Fig. 2:**
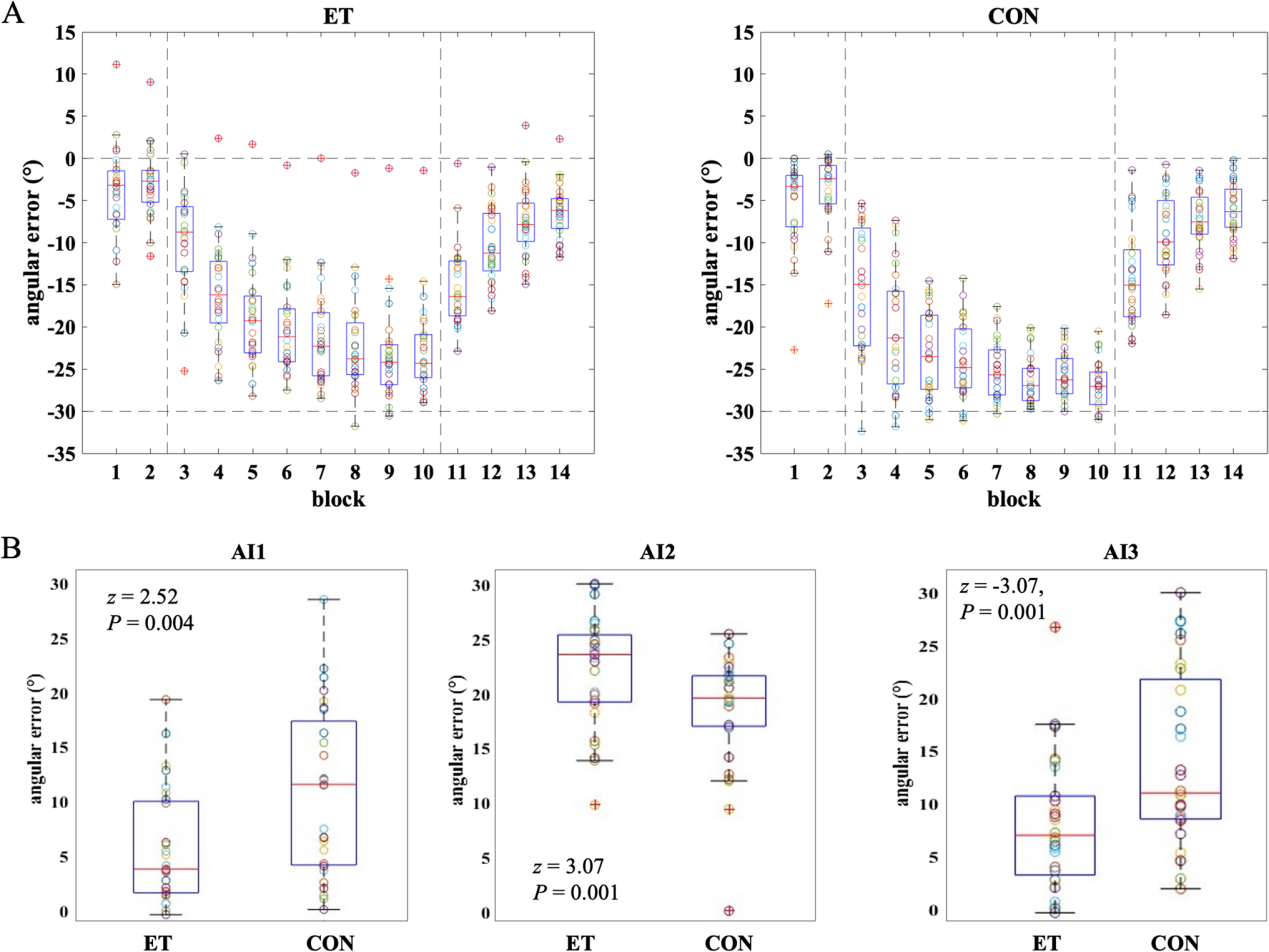
Performance in the visuomotor adaptation task. **A** Distribution of angular errors across individual task blocks (corresponding to Fig. 1F) for each group (ET, Essential Tremor Cohort; CON, control cohort). **B** Adaptation indices (AI) across groups. Controls reached significantly larger angular errors for all AIs, corresponding to better adaptation compared to patients. Note that both AI1 and AI2 are presented here as the absolute of the actual angular error difference (which was negative) for simplicity

Both ET patients and controls were able to adapt and de-adapt to the visual perturbation. Figure 2A illustrates the baseline performance and the change of the angular error after the visual perturbation was introduced and a gradual return to baseline levels when the perturbation was removed. Levels of adaptation and the dynamical course of adaptation between the groups were assessed with two mixed ANOVAs, separately for adaptation (across eight blocks) and de-adaptation (across four blocks). For adaptation, we found a main effect of Group (ET, CON: *F*(1.00) = 11.93, *P* = 0.001), suggesting that adaptation differed between ET patients and controls. A main effect of Block (*F*(2.430) = 132.004, *P* < 0.001) confirmed the change of the angular error with time. The mixed ANOVA did not show significant block by group interactions (*F*(2.430) = 2.05, *P* = 0.122), which means that the rate of adaptation did not differ between the groups. For de-adaptation, a main effect of Block was evident (*F*(2.242) = 164.64, *P* < 0.001) but Group and Block × Group interaction did not reach significance (*P* > 0.1).

To better understand group differences in adaptation dynamics, we examined three predefined phase-specific AIs for the angular error. This allowed us to evaluate individual adaptation compared to the baseline performance and to differentiate between several phases of adaptation (early vs. late adaptation). AI1 is the difference between early adaptation and the second baseline block (ADP1-BL2), AI2 is the difference between late adaptation and the second baseline block (ADP8 – BL2), and AI3 is the difference between de-adaptation and late adaptation DA1 (DA1-ADP8). Better adaptation is therefore evident by stronger negative values in AI1 and AI2 and stronger positive values in AI3. Higher values for AI demonstrate better adaptation. We found significantly larger AIs, in the control group compared to the ET patients (AI1: *z* = 2.52, *P* = 0.004; AI2: *z* = 3.07, *P* = 0.001, AI3: *z* =  − 3.07, *P* = 0.001, FDR corrected for multiple comparisons) (Fig. 2B), suggesting that ET patients had impaired adaptation. Post hoc analysis showed that this impairment was due to deficits in the early adaptation block, ADP1 (*z* = 2.88, *P* = 0.001, FDR corrected) and the late adaptation block ADP8 (*z* = 3.48, *P* = 0.001, FDR corrected). No differences were evident for BL2 (*z* = 0.12, *P* = 0.9) or DA1 (*z* =  − 0.06, *P* = 0.9), which means baseline motor performance and de-adaptation were not impaired in the ET cohort. Notably, an exploratory analysis revealed deficits in the ET group at mid adaptation (ADP4), when compared to the second baseline block (ADP4–BL2: *z* = 2.35, *P* = 0.019). Post hoc analysis showed that this impairment was caused by specific lower angular errors in ET patients compared to control during ADP4 (*z* = 2.74, *P* = 0.006, FDR corrected). There were no group differences in time from movement initiation to peak velocity (all *P* > 0.1), suggesting that movement dynamics in ET patients were not the source for differences in angular errors between the groups. Our results demonstrate that visuomotor adaptation deficits in ET patients were specific to the adaptation phase only.

### No Differences in General Motor Performance between Patients and Healthy Controls

**Fig. 3:**
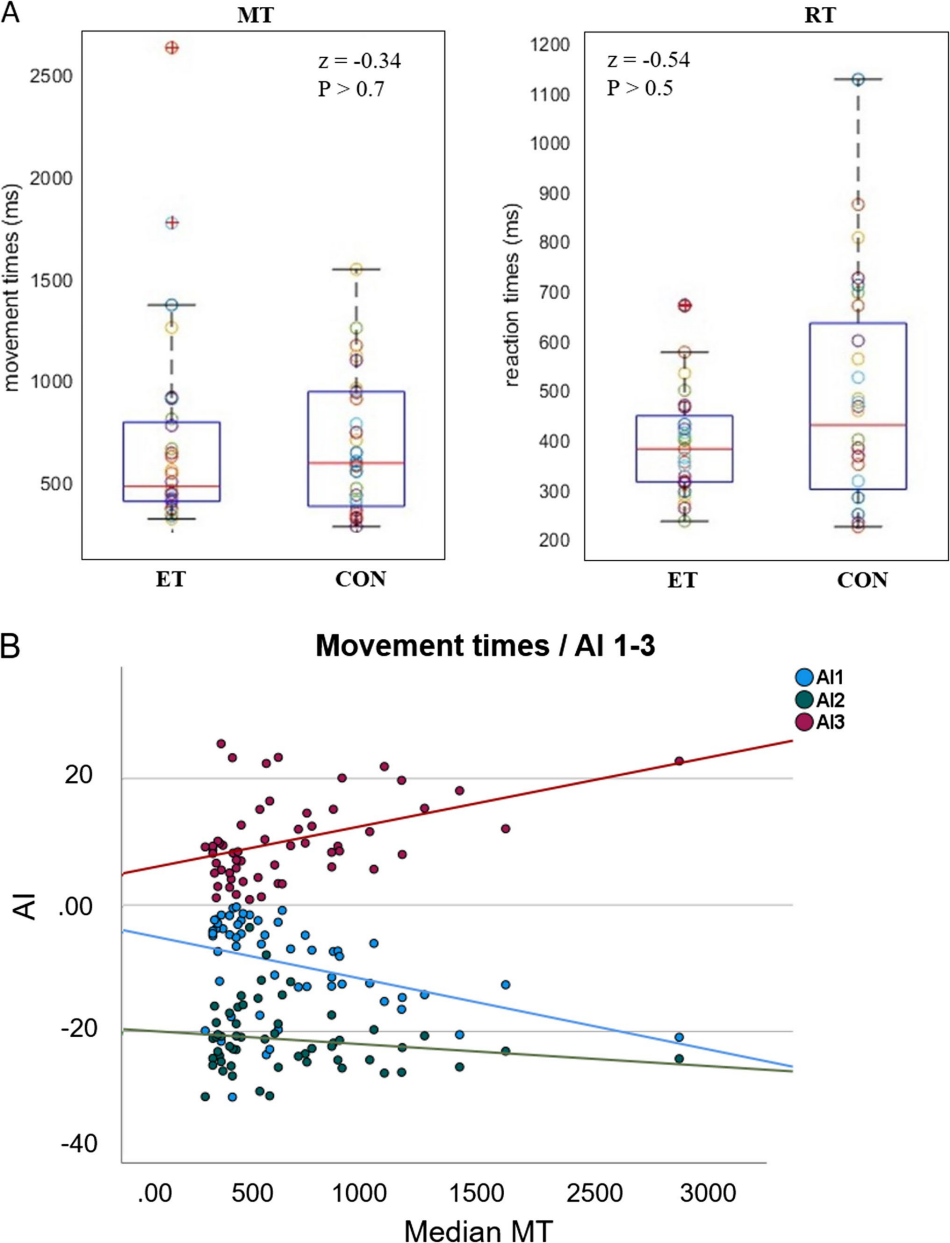
Performance in the visuomotor adaptation task. **A** Box plots for the median movement and reaction times across the groups. No significant differences were observed. **B** Correlation between movement times and the different AIs. All participants (of both groups, *N* = 56) are depicted. Significant correlation between AI1 and AI3 and movement time suggests better adaptation with slower movements

To exclude that visuomotor adaptation deficits were driven by general motor performance changes in ET, we inspected group differences in motor performance by comparing reaction time (RT) and movement time (MT) between the groups. No significant differences were evident for the median MT and median RT (all *P* > 0.5) (Fig. 3A), averaged across all blocks between the groups.

To find whether adaptation was driven by motor performance, we further correlated the median MT of both groups with the different AIs. Significant negative correlation of MT with AI1 (*r* =  − 0.39, *P* = 0.003) and positive correlation with AI3 (*r* = 0.43, *P* = 0.001) (Fig. 3B) suggested that better adaptation was associated with slower movements. Note that excluding two outliers (median MT > 2SD of the groups mean, Fig. 3A) did not influence these relationships (MT/AI1: *r* =  − 0.34, *P* = 0.013; MT/AI3: *r* = 0.36, *P* = 0.007). Post hoc analysis showed that these results were due to a significant correlation with MT during early adaptation (ADP1: *r* =  − 0.33, *P* = 0.016) but not baseline (BL2: *r* =  − 0.08, *P* > 0.5) or de-adaptation (DA1: *r* = 0.18, *P* > 0.1).

These results indicate that group differences in adaptation were not driven by potential differences in general motor performance. Moreover, slower movements during the adaptation and de-adaptation phase were associated with better performance. This effect was probably due to corrective movements.

### No Effect of Pharmacological Therapy on Visuomotor Adaptation

To exclude that visuomotor adaptation deficits were driven by pharmacological therapy, we further tested whether visuomotor adaptation impairments in the ET cohort were still evident when only ET patients without symptomatic pharmacological treatment were included in the analysis. At the time of testing, eight ET patients were on symptomatic disease treatment with propranolol or topiramate (see Supp. Table 1). Twenty patients took no medication to treat ET. Therefore, we compared AIs between patients without symptomatic disease medication and their age-matched healthy controls. Results show still strong differences between patients and controls (AI1: *P* = 0.004, *z* =  − 2.66; AI2: *P* = 0.01, *z* =  − 2.25; AI3: *P* < 0.001, *z* =  − 3.41) (Fig. 3C), suggesting that visuomotor adaptation deficits observed in ET were not due to pharmacological therapy.

### No Associations Between Behavioral Parameters and Clinical Characteristics of Essential Tremor

**Fig. 4:**
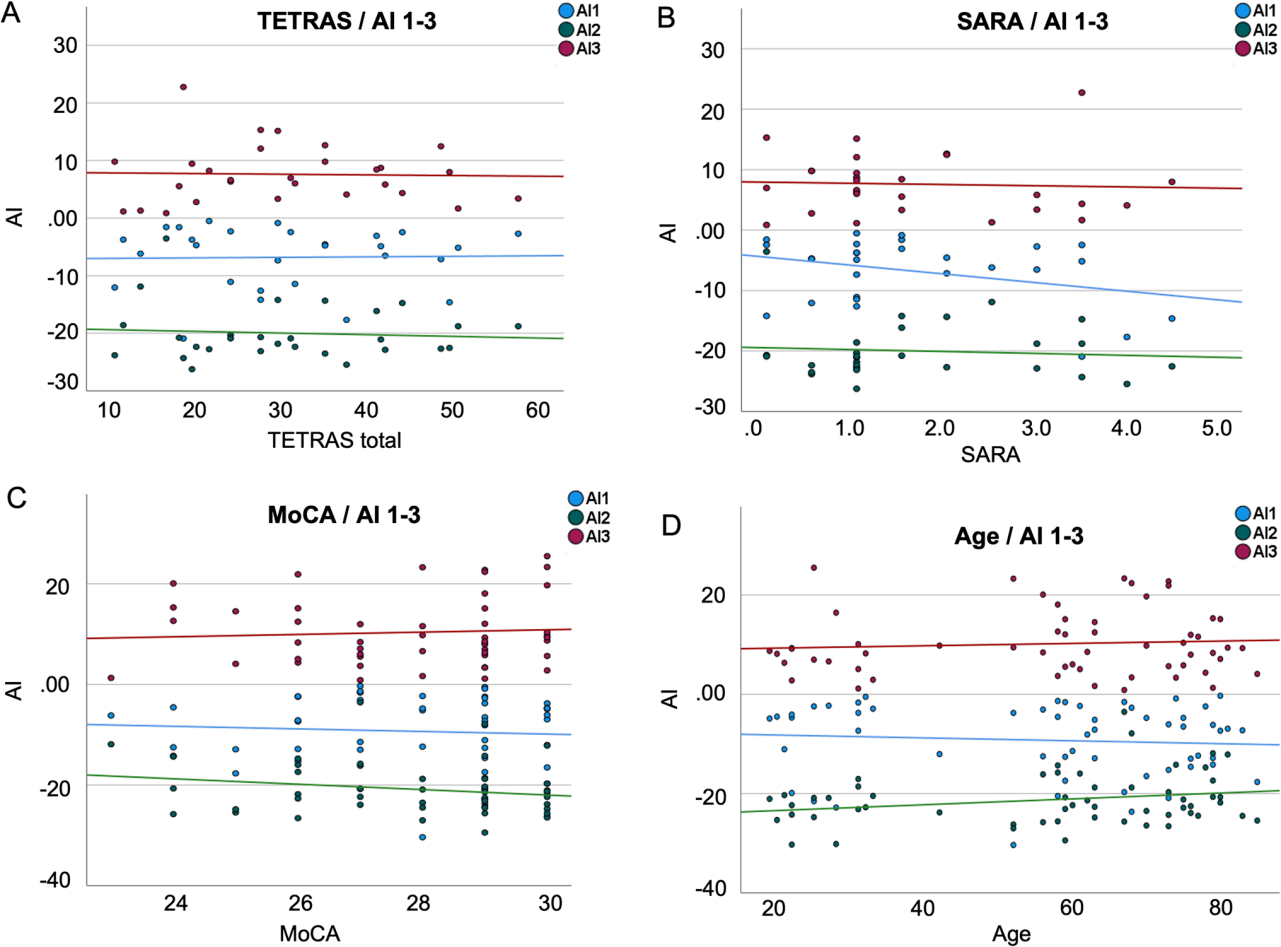
Correlations between clinical characteristics and AIs across all participants. **A** TETRAS = The Essential Tremor Rating Assessment Scale. **B** SARA = Scale for the Assessment and Rating of Ataxia. **C** MoCA = Montreal Cognitive Assessment. **D** Age

Next, we investigated whether visuomotor adaptation was driven by clinical parameters of ET. To this end, we correlated the TETRAS and SARA scores with AIs. We found no association between the SARA score and the different AIs (all* P* > 0.08; Fig. 4B). Note that the SARA score was very low in our ET cohort and did not exceed 5.5 points (out of maximal 40). Furthermore, we found no correlations for TETRAS and AIs, neither for the total score (all *P* > 0.6) (Fig. 4A), nor for the performance subscale (all *P* > 0.6). In addition, no correlation was found between AIs and a summed score of several hand/arm tremor items of TETRAS (finger-nose-test, lateral “wing beating” hold, forward outstretched position and Archimedes spirals, *P* > 0.7). These results indicate that tremor severity as well as very light cerebellar symptoms did not influence visuomotor adaptation.

To explore a possible relationship between tremor severity and motor performance, we further correlated SARA/TETRAS and the median MT in ET patients. No significant correlations were evident (TETRAS: *P* > 0.9, SARA: *P* > 0.2), even when the analysis was confined to the TETRAS hand tremor items (*P* > 0.9). These results indicate that tremor did not influence motor performance in the visuomotor adaptation task.

Due to the progressive nature of ET, we tested for a possible link between disease duration and TETRAS/SARA. No significant results were found for SARA (*P* > 0.3). As expected, strong correlations were evident for disease duration and TETRAS including several TETRAS subscales (TETRAS total: *r* = 0.5, *P* = 0.010; TETRAS Performance subscale: *r* = 0.38, *P* = 0.045; TETRAS ADL subscale: *r* = 0.55, *P* = 0.003).

We then explored whether disease duration was associated with stronger visuomotor adaptation impairment. No significant correlations were found (all AIs: *P* > 0.6), suggesting that disease progression did not lead to progressively stronger impairments in visuomotor adaptation.

### No Effect of Cognitive Decline or Age on Visuomotor Adaptation in Essential Tremor

Given that visuomotor adaptation may also entail a cognitive component, we explored a potential relation between cognitive functions assessed with MoCA and visuomotor adaptation parameters AI1-3. No significant correlations were found (all *P* > 0.1) (Fig. 4C), probably because we only included participants with normal MoCA Scores.

Furthermore, we explored the effect of aging on performance deficits, based on evidence from previous studies showing impairments in elderly subjects. We again found no significant correlation between age and the different AIs (all *P* > 0.3) (Fig. 4D), questioning a general effect of aging on visuomotor adaptation per se.

We then investigated each cohort separately to find whether age affected adaptation differently in ET patients compared to the healthy controls. We found no significant correlation between age and the different AIs in the ET cohort (all *P* > 0.1). In the control cohort, we found a significant correlation between age/AI1 (*r* = 0.46, *P* = 0.038) and age/AI3 (*r* =  − 0.5, *P* = 0.022). Post hoc tests showed that the significance stemmed from a correlation between age and DA1 (*r* =  − 0.56, *P* = 0.009). No significant correlation appeared between age and BL2 or ADP1 (all *P* > 0.1).

## Discussion

In this study, we found that ET patients had deficits in visuomotor adaptation, a capacity that is strongly dependent on the cerebellum. Because alternative explanations for performance deficits could be ruled out, our findings point to cerebellar dysfunction in ET. We found that ET patients were able to perform simple center-target movements, at a precision comparable to healthy volunteers, during the baseline phase prior to the visual perturbation. This finding agrees with previous evidence from studies in cerebellar ataxia patients as well as in patients with cerebellar cortical atrophy [29, 32, 33], in whom reaching accuracy during baseline was not compromised. Furthermore, ET patients’ visuomotor adaptation deficit was not related to clinical characteristics such as tremor or ataxia, as assessed by the TETRAS and the SARA score, nor to disease duration. These findings make impaired visuomotor adaptation due to poor motor coordination an unlikely conclusion. Rather, they suggest that visuomotor adaptation deficits are a genuine feature of ET. In addition, these findings imply that visuomotor adaptation impairments are based on mechanisms independent of those underlying tremor formation or cerebellar ataxia.

Studies investigating which part of the cerebellum is involved in sensorimotor adaptation have utilized functional MRI studies in healthy subjects and voxel-based lesion analysis in neurological patients. In healthy volunteers, ultra-high-field 7 T fMRI revealed that both successful prism adaptation [53], as well as early acquisition of eyeblinks [54], were associated with activation of cerebellar lobule VI [55–57]. Dynamic modulation of cerebellar lobule VI activity was also observed during visuomotor reach adaptation [58]. Individuals with cerebellar stroke or degenerative cerebellar ataxia similarly exhibit impaired eyeblink conditioning [38, 59], force-field adaptation [35, 36], and visuomotor reach adaptation [28, 29, 31, 32, 60, 61]. Although voxel-based morphometry analysis in these patients revealed a contribution of cerebellar crus I and crus II to both force-field and reach adaptation, lesion of lobule VI was specifically associated with impairment of visuomotor reach adaptation [60]. We, therefore, speculate that the impairment of visuomotor adaptation in this study, just as eyeblink conditioning [41] or prism adaptation [42], may be due to dysfunction of lobule VI in ET. Structural analysis of high-resolution MRI images in ET patients has revealed atrophy in posterior cerebellar lobule VIII as well as anterior cerebellar lobules IV and V, but not lobule VI [62]. Therefore, the present findings point to a functional lesion of lobule VI, which is spatially distinct from the cerebellar atrophy pattern of ET patients.

A recent review suggested on the other hand, that lesions to the so-called Guillain-Mollaret (G-M) triangle, known to play a major role in tremor genesis, may affect two main paths connecting to the deep-cerebellar nuclei. The first is a lesion to an excitatory cerebrocerebellar loop, which affects the cerebellar forward model leading to decreased accuracy of prediction and compensation by feedback delay. The second is a lesion to an inhibitory dentato-olivo-cerebellar loop leading to synchronized oscillations in inferior olive neurons [63]. The authors hypothesize that for ET, malfunction in various locations of the G-M triangle is reflected in heterogeneity of clinical ET characteristics including kinetic tremor, intention tremor, and in some cases also rest tremor and the optional appearance of additional “soft neurological signs” like mild cognitive impairment, gait and stance disturbance, or eye-hand-dyscoordination which could also affect visuomotor adaptation abilities [63].

We found evidence for impaired visuomotor adaptation in both early and late adaptation phases, but not during de-adaptation. Patients with ET in the present study could quickly return to the original routine, similar to healthy controls. Interestingly, when transcranial direct stimulation was applied to posterior cerebellar cortex, including lobules VI, crus I and crus II, healthy young and old subjects presented with enhanced adaptation but not de-adaptation [52, 63, 64]. These observations point to different mechanisms of adaptation and de-adaptation. The difference between visuomotor adaptation and de-adaptation could be explained by assuming that the cerebellar region responsible for de-adaptation remains unaffected by cerebellar pathology in ET (or is more resistant to stimulation-induced plasticity). In this case, de-adaptation would only be disrupted if there was more extensive cerebellar pathology. This hypothesis is supported by observations in patients with degenerative cerebellar ataxia in whom cerebellar degeneration is more advanced as compared to patients with ET. Indeed, patients with cerebellar ataxia displayed impairments not only during visuomotor adaptation but also in the de-adaptation phase [32, 33, 58] (although they did express normal de-adaptation in a prism adaptation task [35]). Alternatively, underlying disease mechanisms [65] may differentially affect connectivity patterns within the motor network [15, 62, 66] in ET and cerebellar ataxia. If we accept that lobule VI may play a prominent role in the observed visuomotor adaptation deficits in ET, then the posterior cerebellum might be less important during the process of de-adaptation, when a recall of an already encoded motor routine (simple center-out movements) is required.

We found that advancing age did not negatively affect visuomotor adaptation, even though age was associated with decreased baseline performance in the simple center-out target movements. This is in apparent contrast to previous findings showing that visuomotor adaptation is impaired in older people [67–69]. Because these studies [67–69] did not separately assess baseline performance, movement dysmetria may have erroneously led to the conclusion of impairment of visuomotor adaptation. Because patients and controls were well matched in age in our study, differences in age cannot explain group differences in adaptation.

We also found no association between disease duration and the degree of visuomotor adaptation impairment. Progressive pathological abnormalities in the cerebellum (i.e., loss of Purkinje cells) in ET would likely have led to increasingly severe impairment of visuomotor adaptation [2, 6, 70, 71]. The lack of correlation between duration and impairment of visuomotor adaptation may be seen as indirect support for the hypothesis that ET is not a neurodegenerative disorder [11, 13, 72, 73] but a disease of abnormal neuronal plasticity. However, since assessment of disease duration is inaccurate, if solely based on patients’ self-assessment of first tremor manifestation, clarification of this issue needs further prospective and longitudinal studies [11, 70, 71]. Furthermore, cerebellar atrophy was not correlated with tremor severity in an investigation of structural abnormalities in ET [62].

Impairment of visuomotor adaptation deficits was also present in ET patients without any medication. This observation rules out that deficits in visuomotor adaptability are a result of pharmacological treatment of ET alone. This conclusion is in line with a study [41] reporting that reduced eyeblink acquisition in ET compared to controls was not due to beta-blocker treatment of ET. Medicated patients had stronger impairments of goal-directed movements at baseline compared to the non-medicated patients, suggesting that symptomatic treatment of ET is associated with greater functional impairment or may itself reduce spatial accuracy of goal-directed movements. However, because of the inhomogeneous distribution of participants (nine medicated patients, 25 non-medicated), firm conclusions are not possible.

Although visuomotor adaptation is often regarded as a paradigm of model-based learning, it is now accepted that it also involves model-free learning and explicit strategy learning. Therefore, it could be that cognitive deficits, frequently reported in studies of ET [74–79] and known to be associated with cerebellar pathology, may have driven performance deficits in this task. As no extensive neurocognitive testing was performed in our cohort, and the MoCA test has been shown to have low sensitivity to cerebellar cognitive symptoms [80], an effect of cognitive decline in ET on visuomotor adaptation cannot be ruled out, although patients and healthy controls were comparable in terms of MoCA scores. Since visuomotor reach adaptation is, in turn, often part of everyday motor learning behavior, evidence of its disruption in ET could have significant ecological implications beyond pure motor impairments.

## Conclusions

In a cohort of mildly to moderately affected ET patients, we found evidence for impaired visuomotor adaptation which was not associated with general motor performance, pharmacological therapy, clinical features of tremor, and cerebellar motor symptoms. These results provide evidence of cerebellar dysfunction in ET even in the absence of prominent clinical cerebellar symptoms. Thus, the visuomotor adaptation task may be suitable as a subclinical biomarker of cerebellar dysfunction in ET.

## Supplementary Information

Below is the link to the electronic supplementary material.

Supplementary file1 (DOCX 930 KB)

## Abbreviations

ADP: Adaptation
AI: Adaptation index
BDI: Beck Depression Inventory
BL: Baseline
CON: Controls
DA: De-adaptation
DBS: Deep brain stimulation
EHI: Edinburgh Handedness Inventory
ET: Essential tremor
FDR: False discovery rate
GM: Guillain-Mollaret triangle
MCI: Mild cognitive impairment
MoCA: Montreal Cognitive Assessment
MT: Movement time
RT: Reaction time
SARA: Scale for the Assessment and Rating of Ataxia
SD: Standard deviation
TETRAS: The Essential Tremor Rating Assessment Scale
