# Supplementary material for: Visuomotor Adaptation Deficits in Patients with Essential Tremor
Source: Cerebellum. 2022 Sep 9;22(5):925–37. doi: 10.1007/s12311-022-01474-5 (PMC10485096; doi:10.1007/s12311-022-01474-5)
Supplement: Supplementary file 1 — Supplementary file1 (DOCX 930 KB) [file 12311_2022_1474_MOESM1_ESM.docx]

# Supplementary Materials

**Visuomotor adaptation deficits in patients with Essential Tremor**

Laura Bindel^1^, Christoph Mühlberg^1^, Victoria Pfeiffer^2^, Matthias Nitschke^2^, Annekatrin Müller^1^, Mirko Wegscheider^1^, Jost-Julian Rumpf^1^, Kirsten E. Zeuner^3^, Jos S. Becktepe^3^, Julius Welzel^3^, Miriam Güthe^3^, Joseph Classen^1^, Elinor Tzvi^1,4^

**Author affiliations:**

^1^Department of Neurology, 04103, Leipzig University

^2^Department of Neurology, 23562, University of Lübeck

^3^Department of Neurology, 24105, Kiel University

^4^Syte Institute, 20354, Hamburg

Correspondence to: Dr. Elinor Tzvi-Minker elinortzvi@gmail.com

Dept. of Neurology, Leipzig University, Liebigstraße 20, 04103 Leipzig, Germany

**Table 1: Patients’ characteristics**

| **Patients’ number** | **Age** | **Gender** | **Disease Duration** | **Alcohol Sensitivity** | **disease medication at the time of testing** | **TETRAS** | **SARA** | **MoCA** |
| --- | --- | --- | --- | --- | --- | --- | --- | --- |
| 1 | 80 | Female | 63 | negative | Propranolol | 27.5 | 0 | 24 |
| 2 | 59 | Female | 20 | unknown | Propranolol, Topiramate | 35 | 2 | 24 |
| 3 | 80 | Female | - | unknown | - | 13.5 | 2.5 | 23 |
| 4 | 68 | Female | 3 | negative | - | 16.5 | 0 | 27 |
| 5 | 26 | Male | 9 | positive | - | 31 | 0 | 29 |
| 6 | 76 | Male | 60 | negative | - | 42 | 3 | 29 |
| 7 | 81 | Female | 25 | negative | - | 29.5 | 1 | 26 |
| 8 | 86 | Male | 2 | unknown | - | 37.5 | 4 | 25 |
| 9 | 22 | Female | 6 | positive | - | 24 | 1 | 29 |
| 10 | 91 | Male | 39 | positive | Primidone, Topiramate | 48.5 | 1 | 24 |
| 11 | 78 | Male | 52 | negative | Primidone | 50 | 1 | 27 |
| 12 | 20 | Female | 12 | positive | - | 41.5 | 1 | 30 |
| 13 | 33 | Male | 9 | positive | - | 21.5 | 1 | 29 |
| 14 | 28 | Male | - | unknown | - | 24 | 1 | 28 |
| 15 | 48 | Male | 21 | positive | Primidone | 8 | 0 | 28 |
| 16 | 60 | Male | 10 | positive | - | 18 | 1.5 | 27 |
| 17 | 53 | Male | 4 | unknown | - | 19.5 | 1 | 30 |
| 18 | 32 | Male | 17 | positive | - | 11.5 | 1 | 29 |
| 19 | 74 | Female | 15 | negative | - | 18.5 | 3.5 | 29 |
| 20 | 43 | Male | 1 | positive | - | 10.5 | 0.5 | 30 |
| 21 | 72 | Male | 33 | positive | Primidone, Propranolol | 45.5 | 3 | 24 |
| 22 | 23 | Male | 6 | positive | - | 20 | 0.5 | 30 |
| 23 | 64 | Male | 20 | unknown | - | 48.5 | 2 | 26 |
| 24 | 64 | Female | 54 | positive | - | 50.5 | 3.5 | 28 |
| 25 | 69 | Female | 30 | unknown | - | 57.5 | 3 | 29 |
| 26 | 61 | Male | 46 | unknown | - | 31.5 | 1 | 27 |
| 27 | 79 | Male | 50 | positive | - | 44 | 3.5 | 26 |
| 28 | 77 | Female | 28 | positive | - | 49.5 | 4.5 | 29 |
| 29 | 64 | Male | 7 | negative | Primidone, Propranolol | 32 | 1 | 26 |
| 30 | 77 | Male | 59 | positive | Primidone, Propranolol | 53 | 5.5 | 24 |
| 31 | 71 | Male | 58 | unknown | - | 35 | 0.5 | 28 |
| 32 | 75 | Male | 21 | positive | Propranolol | 29.5 | 1.5 | 29 |
| 33 | 60 | Male | 45 | positive | - | 27.5 | 1 | 29 |
| 34 | 57 | Male | 42 | positive | - | 41 | 1.5 | 27 |

**Section 1-5: Analysis of the entire cohort (including patients who were under primidone treatment)**

1. **Visuomotor adaptation is impaired in Essential Tremor patients**

Both [ET](https://d.docs.live.net/820aedb10610fad6/Desktop/Bindel_Brain_draft_01_11_jjr_jc.docx#_msocom_1) patients and controls were able to adapt and de-adapt to the visual perturbation. Figure 2A illustrates the baseline performance and the change of the angular error after the visual perturbation was introduced and a gradual return to baseline levels when the perturbation was removed. Levels of adaptation and the dynamical course of adaptation between the groups were assessed with two mixed ANOVAs, separately for adaptation (across eight blocks) and de-adaptation (across four blocks). For adaptation, we found a main effect of Group (ET, CON: *F*(1,66) = 10.9, *P* = 0.002), suggesting that adaptation differed between ET patients and controls. A main effect of Block (*F*(7,462) = 173.0, *P* < 0.001) confirmed the change of the angular error with time and a trend for Block x Group interaction (*F*(7,462) = 1.80, *P* = 0.086) suggested that adaptation dynamics differed between the groups. For de-adaptation, a main effect of Block was evident (*F*(7,462) = 191.7, *P* < 0.001) but Group and Block x Group interaction did not reach significance (*P* > 0.1).

We found significantly larger AIs, in the control group compared to the ET patients (AI1: *z* = -2.13, *P* = 0.016; AI2: *z* = -2.28, *P* = 0.01, AI3: *z* = -2.09, *P* = 0.019, FDR corrected for multiple comparisons), suggesting that ET patients had impaired adaptation (Fig. 2B). Post-hoc analysis showed that this impairment was due to deficits in the early adaptation block, ADP1 (*z* = -2.76, *P* = 0.003, FDR corrected) and the late adaptation block ADP8 (*z* = - 3.35, *P* < 0.001, FDR corrected). No differences were evident for BL2 (*z* = -0.50, *P* = 0.3) or DA1 (*z* = - 0.52, *P* = 0.3). Notably, an exploratory analysis revealed deficits in the ET group at mid adaptation (ADP4), when compared to the second baseline block (ADP4 - BL2: *z* = - 2.2, *P* = 0.027). Post-hoc analysis showed that this impairment was caused by specific lower angular errors in ET patients compared to control during ADP4 (Fig. 2A, *z* = - 2.9, *P* = 0.002, FDR corrected).

There were no group differences in time from movement initiation to peak velocity (all *P* > 0.2), suggesting that movement dynamics in ET patients were not the source for differences in angular errors between the groups.


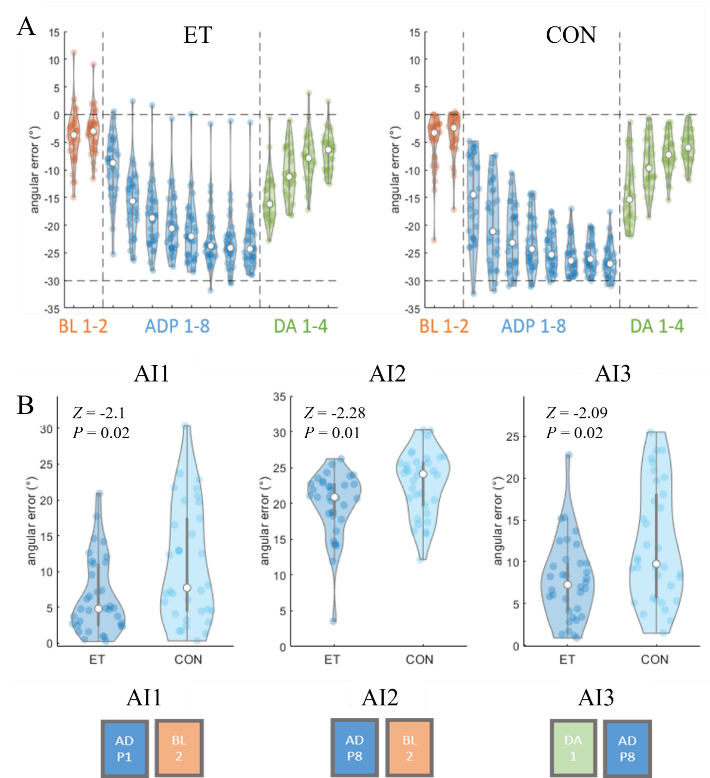


**Fig. 2:** **Performance in the visuomotor adaptation task. (A)** Distribution of angular errors across individual task blocks (corresponding to Fig. 1F) for each group (ET – Essential Tremor cohort, CON – control cohort). **(B)** Adaptation indices (AI) across groups. Controls reached significantly larger angular errors for all AIs, corresponding to better adaptation compared to patients. Note that both AI1 and AI2 are presented here as the absolute of the actual angular error difference (which was negative) for simplicity.

1. **No differences in general motor performance between patients and healthy controls**

No significant differences were evident for the median MT, averaged across all blocks (Fig. 3A), as well as at the different AIs (all *P* > 0.4). The same procedure was performed for the RTs. No significant differences were evident for the median RT, averaged across all blocks (Fig. 3A) between the groups, as well as at the different AIs (all *P* > 0.2).

Significant negative correlation of MT with AI1 (*r* = - 0.40, *P* < 0.001) and positive correlation with AI3 (*r* = 0.40, *P* = 0.001), suggested that better adaptation was associated with slower movements (Fig. 3B). Note that excluding three outliers (median MT > 2SD of the groups mean), did not influence these relationships (MT/AI1: *r* = - 0.35, *P* = 0.002; MT/AI3: *r* = 0.30, *P* = 0.008). Post-hoc analysis showed that these results were due to a significant but rather weak correlation with MT during early adaptation (ADP1: *r* = - 0.16, *P* = 0.002) but not baseline (BL2: *r* = - 0.08, *P* = 0.3) or de-adaptation (DA1: *r* = 0.15, *P* = 0.1).

1. **No effect of pharmacological therapy on visuomotor adaptation**

To exclude that visuomotor adaptation deficits were driven by pharmacological therapy, we further tested whether visuomotor adaptation impairments in the ET cohort were still evident when only ET patients without symptomatic pharmacological treatment were included in the analysis. At the time of testing, nine ET patients were on symptomatic disease treatment with propranolol, primidone and topiramate (see Supp. Table 1). Twenty-five patients took no medication to treat ET. Therefore, we compared AIs between patients without symptomatic disease medication and their age-matched healthy controls. Results show still strong differences between patients and controls (AI1: *P* = 0.004, *z* = - 2.66; AI2: *P* = 0.01, *z* = -2.25; AI3: *P* < 0.001, *z* = - 3.41) (Fig. 3C), suggesting that visuomotor adaptation deficits observed in ET were not due to pharmacological therapy.

Despite the small sample size, we explored in the medicated ET cohort (*n* = 9) whether pharmacological therapy influenced visuomotor adaptation, when comparing those patients with the non-medicated patients (*n* = 25). Whereas this analysis revealed no significant differences for AI1 and AI3 (*P* > 0.1), AI2 was found to be significantly different (*P* = 0.03, *z* = - 1.89), indicating that the non-medicated patients reached better end-performance compared to the medicated patients (Fig. 3C). Post-hoc tests revealed that significance stemmed from baseline differences between the groups (BL2: *z* = - 2.36, *P* = 0.009) and not from late adaptation differences (ADP8: *P* > 0.3), indicating that pharmacotherapy to treat ET may have affected simple target reaching performance but not visuomotor adaptation.


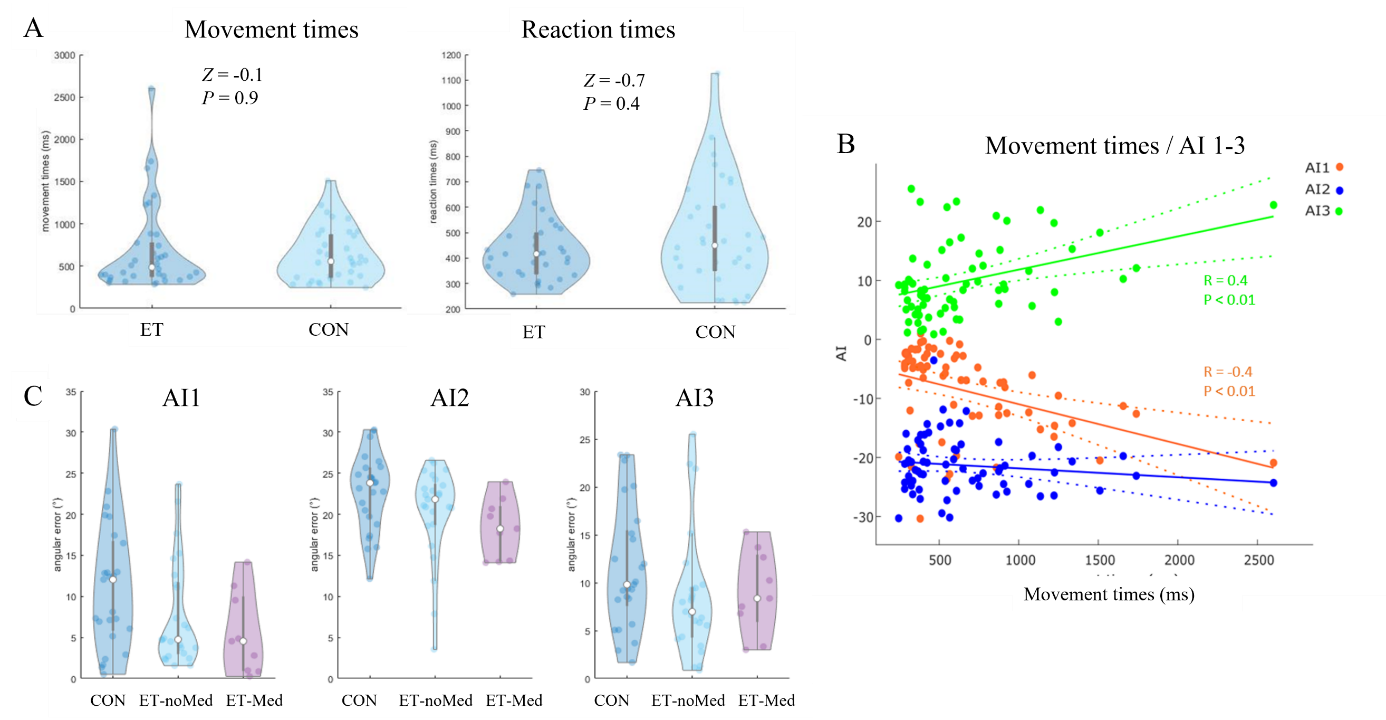


**Fig. 3: Performance in the visuomotor adaptation task.** (**A**) Violin plots for the median movement and reaction times across the groups. No significant differences were observed. (**B**) Correlation between movement times and the different AIs. All participants (of both groups, N = 68) are depicted. Significant correlation between AI1 as well as AI3 and movement time suggests better adaptation with slower movements. (**C**) The effect of medication on adaptation. Depicted are the different AIs for the control cohort (CON: N = 34), the medicated patient cohort (ET-Med: N = 9) and the non-medicated patient cohort (ET-noMed: N = 25). Controls performed significantly better compared to the non-medicated patients (AI1: *P* = 0.004; AI2: *P* = 0.01; AI3: *P* < 0.001). At AI2, non-medicated patients performed better compared to medicated patients (*P* = 0.03).

1. **No associations between behavioral parameters and clinical characteristics of Essential Tremor**

Next, we investigated whether visuomotor adaptation was driven by clinical parameters of ET. To this end, we correlated the TETRAS and SARA scores with AIs. We found no association between the SARA score and the different AIs (Fig. 4B). Furthermore, we found no correlations for TETRAS and AIs, neither for the total score (all *p* > 0.2) (Fig. 4A), nor for the performance subscale (all *P* > 0.1). In addition, no correlation was found between AIs and a summed score of several hand/arm tremor items of TETRAS (finger-nose-test, lateral “wing beating” hold, forward outstretched position and Archimedes spirals, *P* > 0.1).

We further correlated SARA/TETRAS and the median MT in ET patients. No significant correlations were evident (TETRAS: *P* > 0.4, SARA: *P* > 0.1), also when the analysis was confined to the TETRAS hand tremor items (*P* > 0.3).

We tested for a possible link between disease duration and TETRAS/SARA. No significant results were found for SARA (*r* = 0.20, *P* = 0.1). As expected, strong correlations were evident for disease duration and TETRAS including several TETRAS subscales (TETRAS total: *r* = 0.59, *P* = 0.001; TETRAS Performance subscale: *r* = 0.41, *P* = 0.012; TETRAS hand tremor items: *r* = 0.32, *P* = 0.042).

We then explored whether disease duration was associated with stronger visuomotor adaptation impairment. No significant correlations were found (all AIs: *P* > 0.3).

1. **No effect of cognitive decline or age on visuomotor adaptation in Essential Tremor**

Given that visuomotor adaptation may also entail a cognitive component, we explored a potential relation between cognitive functions assessed with MoCA and visuomotor adaptation parameters AI1-3. While no significant correlations were found in AI3 (*r* = 0.14, *P* = 0.1), a tendency for a negative correlation in AI1 (*r* = - 0.18, *P* = 0.08) and a significant negative correlation in AI2 (*r* = - 0.301, *P* = 0.009, FDR corrected), suggested that stronger cognitive impairments led to worse adaptation (Fig. 4C). Note, however, that post-hoc tests showed that this correlation stemmed from better performance with higher MoCA at baseline (BL2: *r* = 0.22, *P* = 0.04) while no correlations were evident during late adaptation (ADP8: *r* = - 0.13, *P* = 0.17), stressing the general effect of cognitive decline on accuracy in target reaching rather than on visuomotor adaptation per se.

Finally, we explored the effect of aging on performance deficits, based on evidence from previous studies showing impairments in elderly subjects. We found no significant correlation between age and AI1 and AI3 across participants of both groups (all *P* > 0.4), however AI2 significantly correlated with age (*P* = 0.005, *r* = 0.31, Fig. 4D). Similar to the MoCA effects reported above, post-hoc tests showed that this effect stemmed from a significant correlation in the second baseline block (*r* = - 0.28, *P* = 0.01) but not late adaptation (ADP8: *P* = 0.2). We then investigated each cohort separately to find whether age affected adaptation differently in ET patients compared to the healthy controls. We found a positive correlation between age and AI2 in the control group (*r* = 0.39, *P* = 0.012) but not in the ET group (*P* > 0.09). Post-hoc tests showed here as well that significance stemmed from a correlation at the second baseline block (*r* = - 0.32, *P* = 0.03) and not at late adaptation (ADP8: *P* > 0.1), stressing the effect of aging on accuracy in target reaching rather than on visuomotor adaptation per se.


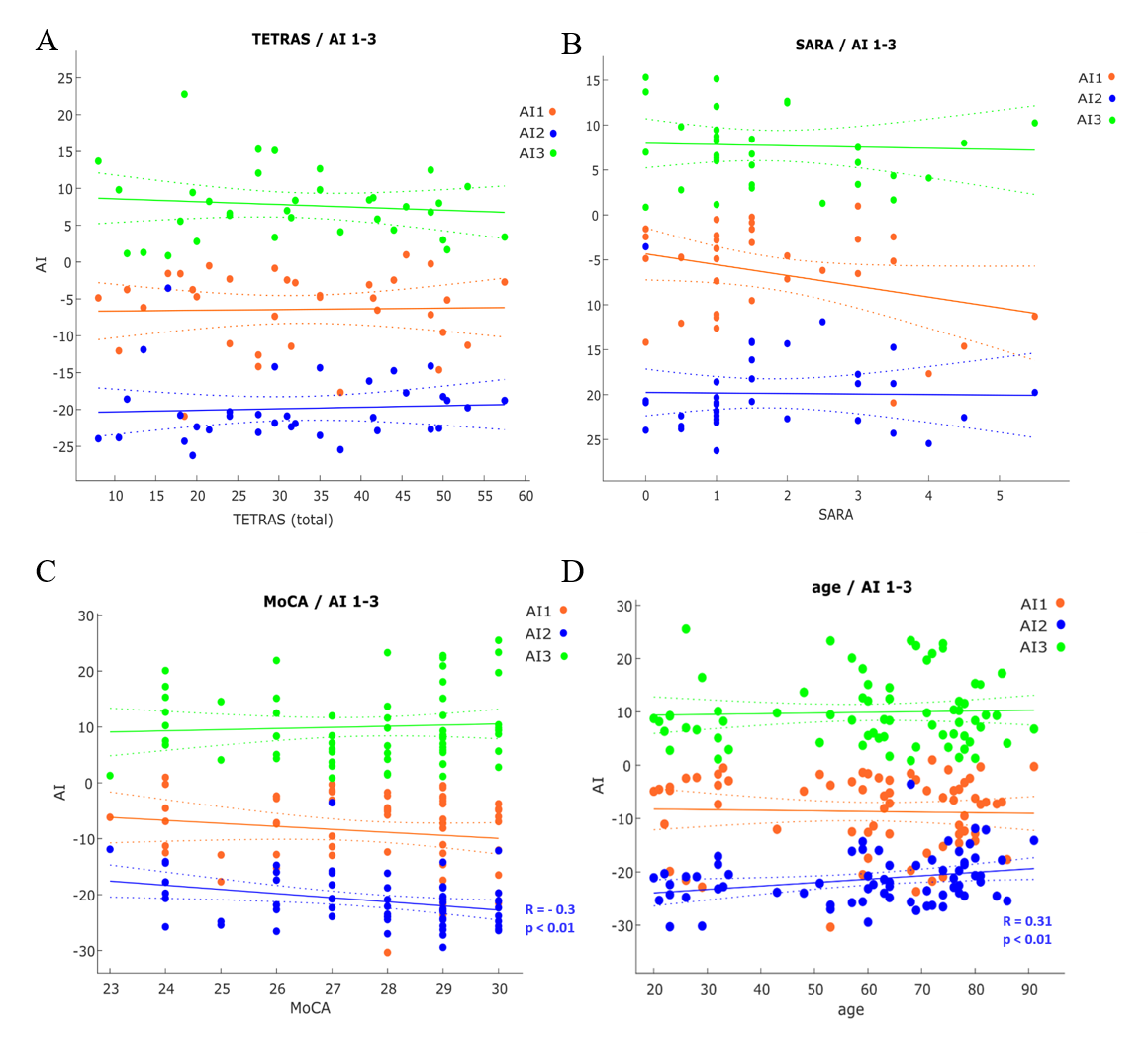

**Fig. 4: Correlations between clinical characteristics and AIs across all participants. (A)** TETRAS = The Essential Tremor Rating Assessment Scale. **(B)** SARA = Scale for the Assessment and Rating of Ataxia. **(C)** MoCA = Montreal Cognitive Assessment. **(D)** Age.
